# Extracapsular Dissection Versus Traditional Parotid Surgery: A Comprehensive Review of Techniques and Outcomes

**DOI:** 10.7759/cureus.69141

**Published:** 2024-09-10

**Authors:** Saurabh Gawand, Rajesh G Gattani, Chava Aravind Kumar, Apoorva Pande

**Affiliations:** 1 General Surgery, Jawaharlal Nehru Medical College, Datta Meghe Institute of Higher Education and Research, Wardha, IND; 2 Pathology, Jawaharlal Nehru Medical College, Datta Meghe Institute of Higher Education and Research, Wardha, IND

**Keywords:** extracapsular dissection, facial nerve injury, parotid tumours, surgical outcomes, traditional parotid surgery, tumour recurrence

## Abstract

Parotid tumours, encompassing both benign and malignant forms, present significant challenges in surgical management. Traditional parotid surgery, including various forms of parotidectomy, has long been the standard approach, aiming for complete tumour removal while addressing potential complications such as facial nerve injury. However, extracapsular dissection (ECD) has emerged as an alternative technique, focusing on excising the tumour along with a thin layer of surrounding tissue, which may offer benefits in preserving healthy glandular tissue and reducing postoperative complications. This review comprehensively compares ECD and traditional parotid surgery techniques, evaluating their efficacy, outcomes, and associated complications. We analyse clinical studies and evidence to assess differences in tumour recurrence rates, facial nerve function preservation, and overall patient recovery. Additionally, the review explores the indications for each surgical approach, considering tumour characteristics and patient-specific factors. The findings suggest that while ECD may offer advantages in terms of reduced postoperative complications and improved preservation of glandular tissue, traditional parotidectomy remains a robust method for managing complex cases. This review aims to inform clinical decision-making by presenting a detailed comparison of both techniques, ultimately guiding surgeons in selecting the most appropriate approach for individual patients.

## Introduction and background

Parotid tumours are neoplasms in the parotid glands, the largest salivary glands near the jawline [1]. These tumours are classified into benign and malignant types. Benign parotid tumours, such as pleomorphic adenomas, are more prevalent and generally present less aggressive clinical behaviour [1]. Despite being non-cancerous, they can cause significant symptoms and complications due to their size and proximity to vital structures. On the other hand, malignant parotid tumours, including mucoepidermoid carcinoma, adenoid cystic carcinoma, and squamous cell carcinoma, are less common but pose a more severe threat due to their aggressive nature and potential for metastasis [2]. The management of these tumours often involves surgical intervention, which is crucial for both complete tumour removal and minimising the risk of recurrence [2].

Surgical management is pivotal in the treatment of parotid tumours, aiming to achieve effective tumour removal while preserving as much healthy tissue as possible. Traditional surgical techniques typically involve various forms of parotidectomy, where the tumour and a portion of the parotid gland are removed [3]. However, the introduction of extracapsular dissection (ECD) presents an alternative approach. ECD focuses on excising the tumour and a thin layer of surrounding tissue to preserve more healthy glandular tissue and reduce postoperative complications. The choice of surgical technique significantly impacts patient outcomes, including the risk of facial nerve injury and the overall recovery process [4].

This review aims to comprehensively compare ECD and traditional parotid surgery techniques. The review highlights differences in surgical methods, indications, and outcomes by evaluating both approaches. Furthermore, it will assess the relative efficacy of each technique in terms of tumour removal, recurrence rates, and postoperative complications. This analysis will be grounded in the latest clinical evidence and studies, offering insights into which technique may offer better outcomes for patients with parotid tumours. The ultimate goal is to guide clinicians in choosing the most appropriate surgical strategy based on current data and patient-specific factors.

## Review

Parotid tumour surgery: Historical perspective

The surgical management of parotid tumours has evolved significantly since the early 20th century. Initially, the primary approach was tumour enucleation, which involved removing the tumour without formally exposing the facial nerve. This technique, prevalent until the mid-20th century, was associated with high recurrence rates, particularly for pleomorphic adenomas, frequently misclassified as hamartomas rather than true neoplasms [5]. By the 1920s, there was increasing recognition of the importance of preserving the facial nerve during surgery. Surgeons like Carwardine and Sistrunk began refining techniques that involved wider dissection and careful identification of the facial nerve. The development of the pre-auricular incision provided improved access and visibility, which was crucial for safeguarding the nerve [6]. A pivotal shift occurred in the 1950s with the recognition that more extensive surgical procedures could reduce recurrence rates. Surgeons increasingly adopted superficial parotidectomy (SP), which involved removing the superficial lobe of the parotid gland while preserving the facial nerve. This technique was notably advanced by Patey and Thackray, whose influential work emphasised the critical role of exposing and preserving the facial nerve during surgery [7]. The introduction of ECD marked a major advancement in the surgical treatment of benign parotid tumours. ECD involves removing the tumour and a thin rim of surrounding glandular tissue, reducing the risk of both facial nerve damage and tumour recurrence. This technique emerged in response to the limitations of traditional methods, particularly concerning complications and cosmetic outcomes [8]. ECD gained popularity in the late 20th and early 21st centuries as surgeons sought less invasive options that still provided effective oncological control. Research has demonstrated that ECD can achieve recurrence rates comparable to traditional approaches while offering reduced morbidity and improved aesthetic outcomes. This technique is especially advantageous for small, superficial tumours, as it allows for quicker recovery and less postoperative discomfort compared to more extensive surgeries like SP [9].

ECD

ECD is an innovative surgical technique that removes benign parotid tumours. This method involves excising the tumour along with a thin rim of surrounding normal tissue while avoiding formal facial nerve dissection. The procedure begins with a conservative skin incision tailored to the tumour's size and location. Surgeons then expose the parotid gland capsule and palpate the tumour to confirm that it is well-defined and does not infiltrate the surrounding tissues [4]. A cruciate incision is made over the tumour, extending approximately 1 cm beyond its margins. The parotid fascia and underlying tissue are carefully retracted, allowing for a meticulous blunt dissection through the gland until the tumour is reached. The tumour is then excised along with a thin layer of surrounding tissue to minimise the risk of nerve damage [10]. ECD is particularly suited for small, superficial, and mobile benign parotid tumours that are well-circumscribed and do not involve the facial nerve. Proper patient selection is critical, typically involving preoperative imaging such as ultrasound or MRI, along with a fine-needle aspiration (FNA) to confirm the benign nature of the tumour. ECD may not be appropriate for patients with larger tumours or those suspected of malignancy, as the risk of incomplete resection increases [11]. The benefits of ECD are substantial. Chief among them is the reduced risk of facial nerve injury, a common concern in traditional parotid surgery. Additionally, ECD has been associated with lower rates of Frey's syndrome, a condition marked by sweating in the cheek area during eating, which often occurs after conventional parotidectomy. The technique also tends to result in shorter operative times and faster patient recovery [12]. However, potential limitations exist, including the risk of incomplete tumour removal, which could lead to recurrence, and the possibility of tumour seeding during the procedure. In some instances, if the tumour proves to be more extensive than initially anticipated, the surgeon may need to convert to a more comprehensive parotidectomy [13]. Recent studies provide strong evidence supporting ECD as a viable alternative to traditional SP for benign parotid tumours. Meta-analyses have demonstrated that ECD achieves comparable oncological outcomes with similar recurrence rates. Notably, one study found that ECD resulted in lower facial nerve paresis and Frey's syndrome incidences than SP [14]. Furthermore, long-term data show that the five-year and 10-year cancer-specific survival rates for clinically benign tumours treated with ECD are 100% and 98%, respectively. While these outcomes are promising, continued long-term follow-up is necessary to ensure the durability of these results [15].

Traditional parotid surgery

**Figure 1:**
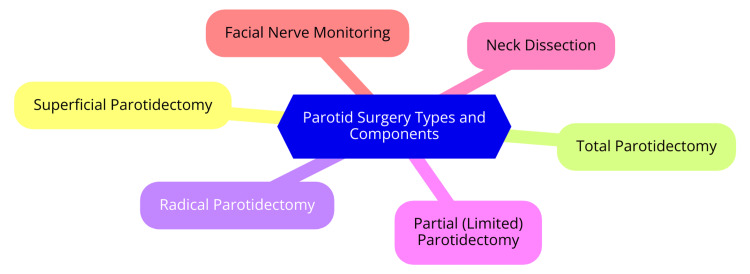
Key types and components of traditional parotid surgery Image Credit: Dr Saurabh Gawand

Traditional parotid surgery, commonly referred to as parotidectomy, involves the surgical removal of part or all of the parotid gland. The most widely used approach is the Modified Blair incision, which provides extensive access to the parotid gland and nearby structures, including the facial nerve [16]. Over time, variations such as the facelift incision have been developed to reduce visible scarring and enhance aesthetic outcomes while offering adequate exposure to the facial nerve and surrounding tissues [17]. There are two primary forms of traditional parotid surgery: SP and total parotidectomy. SP involves removing only the superficial lobe of the parotid gland, where most benign tumours, such as pleomorphic adenomas, are typically located. This approach is favoured when the tumour is well-circumscribed and does not extend into deeper structures [18]. In contrast, total parotidectomy entails removing the entire parotid gland. It is generally reserved for malignant tumours or more extensive diseases, requiring meticulous dissection to preserve the facial nerve and adjacent structures [3]. Patients suitable for traditional parotid surgery include those with confirmed benign tumours that necessitate removal to prevent a recurrence, individuals with malignant tumours requiring complete excision, and those with tumours causing symptoms or cosmetic concerns. Preoperative evaluations, such as imaging and biopsy, are critical for assessing tumour characteristics and determining the optimal surgical strategy [19]. Traditional parotid surgery offers several advantages, including effective tumour removal, reduced risk of recurrence, and a well-established protocol with a long history of successful outcomes. However, it also presents challenges, such as an increased risk of facial nerve injury, longer recovery periods, and aesthetic concerns due to more noticeable scarring [18]. Numerous studies have assessed the outcomes of traditional parotidectomy. Research shows that SP has a low recurrence rate for benign tumours, though it is associated with complications such as Frey's syndrome and facial nerve damage. A meta-analysis reported recurrence rates for pleomorphic adenomas following SP to be around 1-5% [20]. In malignant tumours, total parotidectomy, especially when combined with adjuvant therapies, can result in favourable survival outcomes. Long-term follow-up studies indicate that patients with early-stage malignancies experience significantly improved survival rates after surgical excision [21]. Key types and components of traditional parotid surgery are illustrated in Figure 1.

Comparative analysis

Surgical Outcomes

When comparing the efficacy of ECD and SP, both techniques show comparable outcomes in terms of complete tumour removal and recurrence rates [22]. Research indicates that recurrence rates for benign parotid tumours are similar between the two methods, with ECD achieving low recurrence rates without compromising oncological safety [12]. A systematic review highlighted that ECD could offer similar recurrence outcomes to SP while minimising surgical complications, making it a viable option for managing benign tumours [12]. However, the complication rates associated with these approaches differ significantly. ECD is associated with a lower incidence of temporary facial nerve injury, with only about 4% of patients experiencing this complication compared to approximately 20% of those undergoing SP [23]. Additionally, hematoma formation is less frequent in ECD patients, occurring in 3.9% of cases compared to 15.3% for SP. Frey’s syndrome, a common postoperative complication after SP, also occurs much less often following ECD, with rates of about 5% compared to 32% for SP. ECD presents a more favourable profile concerning complications, particularly in preserving facial nerve function and reducing postoperative morbidities [24].

Quality of Life and Functional Outcomes

Patients undergoing ECD generally experience faster recovery and an improved quality of life following surgery. The average hospital stay for ECD is significantly shorter, around 0.5 days, compared to approximately 1.3 days for SP [25]. This quicker recovery is largely due to the minimally invasive nature of ECD, which results in fewer complications and enables patients to resume normal activities more swiftly. As a result, many patients report higher satisfaction with their postoperative experience [26]. Regarding functional outcomes, ECD demonstrates distinct advantages, particularly concerning facial nerve preservation and salivary function. Temporary facial nerve dysfunction is significantly lower among ECD patients, which is essential for maintaining facial aesthetics and motor function [27]. Furthermore, the likelihood of developing gustatory sweating, or Frey’s syndrome, is reduced after ECD, contributing to better overall patient satisfaction. These factors collectively enhance the quality of life for individuals undergoing ECD, making it a highly attractive option for the surgical management of benign parotid tumours [28].

Cost-Effectiveness

From a cost-effectiveness standpoint, ECD has proven more economical than SP. Overall hospital expenses, including those for anaesthesia and operating room time, are significantly lower for ECD, primarily due to shorter procedure times and reduced hospital stays [29]. ECD procedures take approximately 83.5 minutes, compared to 139 minutes for SP, leading to decreased anaesthesia duration and overall hospital costs. This financial benefit is particularly important in healthcare systems facing increasing demand for surgical interventions. The economic impact of adopting ECD over SP is especially significant in resource-limited settings. The reduced costs associated with ECD help alleviate the financial burden on patients while optimising the utilisation of healthcare resources. This efficiency is vital in managing the rising incidence of benign parotid tumours and ensuring that healthcare resources are deployed effectively [30]. ECD represents a highly attractive alternative to traditional SP for benign parotid tumours, offering comparable oncological outcomes with fewer complications, improved quality of life, and enhanced cost-effectiveness. These advantages make ECD an increasingly preferred approach in modern surgical practice [31].

Current guidelines and recommendations

Review of Clinical Guidelines

Recent guidelines from professional societies, such as the American Head and Neck Society (AHNS) and the European Society of Medical Oncology (ESMO), offer crucial frameworks for managing parotid tumours, particularly regarding surgical approaches and patient selection [32]. The AHNS underscores the importance of accurate preoperative diagnosis through imaging and FNA before surgical intervention. Their guidelines suggest that benign tumours, including pleomorphic adenomas and Warthin's tumours, can be effectively treated with either SP or ECD, depending on the tumour's characteristics and the surgeon's expertise [33]. Similarly, ESMO advocates a multidisciplinary approach to managing salivary gland tumours, emphasising the need for individualised treatment plans based on tumour type, size, and location. ESMO endorses using ECD for select benign tumours, particularly those that are small, superficial, and mobile, as this technique reduces the morbidity associated with more traditional surgical methods [34].

Recommendations Based on Review

ECD may be the preferred surgical technique in certain scenarios, especially for small, well-defined, and superficial benign tumours. Tumours such as pleomorphic adenomas and Warthin's tumours, which are encapsulated and do not infiltrate surrounding tissues, are ideal candidates for ECD due to the lower risk of complications associated with this approach [11]. This technique is also particularly beneficial for patients who are at higher risk of complications from traditional surgery, such as older individuals or those with significant comorbidities, as it is less invasive and allows for a quicker recovery [35]. When determining the most appropriate surgical approach, it is essential for surgeons to carefully assess tumour characteristics, including size, location, and histological type. Encapsulated, superficial tumours are more suitable for ECD, while larger or infiltrative tumours may require a more extensive procedure like SP. Additionally, the surgeon's expertise is a critical factor in the procedure's success, as ECD demands a deep understanding of both the technique and the intricate anatomy of the parotid region [36]. A thorough preoperative evaluation, including imaging studies and FNA, is crucial for determining the best surgical strategy. The decision-making process should involve a comprehensive discussion with the patient, addressing each approach's potential risks and benefits. Current guidelines advocate for a personalised approach to managing parotid tumours, with ECD increasingly recognised as a viable option for select benign cases, particularly when reducing morbidity is a priority [37]. Table 1 outlines current guidelines and recommendations for the surgical management of parotid tumours.

**Table 1:** Current guidelines and recommendations for surgical management of parotid tumours

Organisation	Guideline/Recommendation	Surgical Approach	Indications/Criteria	Postoperative Management
American Head and Neck Society (AHNS) [4]	Extracapsular dissection (ECD) is recommended for small, benign, and mobile parotid tumours.	ECD	Tumours <4 cm, no facial nerve involvement	Routine follow-up with MRI/CT every 6-12 months
European Society for Medical Oncology (ESMO) [8]	Advocates ECD for low-grade benign tumours, while traditional surgery is recommended for larger or malignant tumours.	ECD, Superficial Parotidectomy	Small, benign tumours, high-risk tumours (traditional surgery)	Regular clinical examination and imaging
National Comprehensive Cancer Network (NCCN) [38]	Recommends traditional surgery for malignant parotid tumours, particularly those involving facial nerve preservation.	Superficial or Total Parotidectomy	Malignant tumours, tumours >4 cm, facial nerve involvement	Radiotherapy in high-risk cases, follow-up imaging
British Association of Head and Neck Oncologists (BAHNO) [8]	Suggests ECD for benign lesions without facial nerve complications and traditional surgery for malignancies.	ECD, Traditional Parotidectomy	Benign tumors (ECD), Malignant tumors (Traditional Surgery)	Monitoring with imaging, potential adjuvant therapy
American Society of Clinical Oncology (ASCO) [8]	Advises traditional surgery for high-risk patients and larger tumours, while ECD is acceptable for benign, smaller tumours.	ECD, Total or Superficial Parotidectomy	Benign tumours <4 cm, malignant or recurrent tumours	Surveillance and optional adjuvant radiotherapy for malignancies

Future directions and research

Recent advancements in parotid surgery have focused on enhancing outcomes through innovative techniques. One such advancement is minimally invasive approaches, particularly endoscopic parotidectomy. This technique minimises scarring and improves recovery using smaller incisions and advanced visualisation tools. Gasless endoscopic parotidectomy, which uses a single incision plus technique, has shown promise in preserving facial nerve function while effectively excising tumours. Future research should focus on refining these methods and exploring their applicability to larger or deeper tumours [39]. Beyond surgical techniques, there is a growing need for long-term studies and larger clinical trials to evaluate the effectiveness and sustainability of emerging approaches like ECD. While early studies suggest that ECD offers comparable outcomes to SP in terms of recurrence rates and complications, long-term follow-up is essential to verify the durability of these results. This research will ensure that newer approaches provide consistent, reliable patient outcomes [40]. Technological advancements such as robotic-assisted surgery and enhanced imaging modalities also promise to transform parotid surgery. These tools can improve precision, reduce surrounding tissue trauma, and minimise postoperative complications. As these technologies are refined, they could become standard practice for a broader spectrum of parotid tumours, including those traditionally requiring more invasive surgery [41]. Improvements in postoperative care and rehabilitation are equally important for optimising recovery. Enhanced rehabilitation protocols, including physical therapy for facial nerve function and management strategies for Frey's syndrome, could significantly improve patient outcomes. In addition, patient education programs that inform patients about postoperative expectations and self-care strategies can empower them, enhancing satisfaction with their surgical results [42]. By integrating advancements in surgical techniques and postoperative care, the future of parotid surgery is poised for significant improvements, resulting in better patient experiences and outcomes. Key areas of future research and innovation in ECD and traditional parotid surgery are summarised in Table 2.

**Table 2:** Key areas of future directions and research in extracapsular dissection (ECD) and traditional parotid surgery

Focus Area	Description	Potential Impact
Long-Term Outcome Studies [43]	Conducting studies to assess long-term tumour recurrence, survival rates, and quality of life post-surgery.	Better understanding of the durability and success of ECD vs. traditional surgery.
Minimally Invasive Techniques [44]	Exploration of less invasive surgical methods, possibly enhancing ECD with advanced imaging or robotic assistance.	Reduced recovery time, fewer complications, and better aesthetic outcomes.
Facial Nerve Preservation [45]	Research into methods and technologies to improve the protection of the facial nerve during surgery.	Reduced incidence of facial nerve damage and improved patient functional outcomes.
Cost-Effectiveness Analyses [46]	Evaluating the economic implications of ECD versus traditional surgery, including resource use and recovery.	Informed decision-making in healthcare resource allocation and patient care planning.
Technological Integration [46]	Integration of intraoperative tools (e.g., nerve monitoring, 3D imaging) to improve surgical precision.	Increased safety, better precision, and fewer surgical complications.
Personalised Surgical Planning [47]	Development of tailored surgical approaches based on tumour type, location, and patient characteristics.	Improved patient outcomes with individualised, targeted surgical strategies.
Combination With Adjunct Therapies [48]	Investigating the role of adjuvant therapies (e.g., radiotherapy) in conjunction with ECD and traditional surgery.	Enhanced treatment outcomes, especially in malignant parotid tumours.
Comparative Trials [49]	Large-scale randomised controlled trials comparing ECD and traditional surgery in varied tumour settings.	High-quality evidence to guide surgical decision-making and best practice standards.

## Conclusions

In conclusion, managing parotid tumours through surgical intervention requires careful consideration of the techniques employed to ensure optimal outcomes. ECD and traditional parotid surgery each offer distinct advantages and limitations. ECD provides a less invasive approach that may reduce the risk of complications and preserve healthy glandular tissue, potentially leading to improved functional outcomes and a lower recurrence rate. Conversely, traditional parotidectomy remains a well-established method that offers thorough tumour removal but may come with a higher risk of facial nerve injury and longer recovery time. By comparing these techniques, this review underscores the importance of individualised surgical planning, considering tumour characteristics and patient factors to select the most appropriate approach. Future research and clinical trials will be essential in refining these techniques and enhancing our understanding of their long-term outcomes. Ultimately, the choice of surgical method should balance effective tumour management with preserving the quality of life, ensuring that patients receive the best possible care based on the latest evidence and evolving clinical practices.
